# A genus-level taxonomic review of primitively segmented spiders (Mesothelae, Liphistiidae)

**DOI:** 10.3897/zookeys.488.8726

**Published:** 2015-03-21

**Authors:** Xin Xu, Fengxiang Liu, Jian Chen, Hirotsugu Ono, Daiqin Li, Matjaž Kuntner

**Affiliations:** 1 Centre for Behavioural Ecology and Evolution (CBEE), and Hubei Collaborative Innovation Center for Green Transformation of Bio-Resources, College of Life Sciences, Hubei University, Wuhan, 430062, China; 2 Department of Biological Sciences, National University of Singapore, Singapore; 3 Evolutionary Zoology Laboratory, Biological Institute ZRC SAZU, Ljubljana, Slovenia; 4 Department of Entomology, National Museum of Natural History, Smithsonian Institution, Washington, D.C, USA; 5 Department of Zoology, National Museum of Nature and Science, 4-1-1 Amakubo, Tsukuba-shi, Ibaraki-ken, 305-0005, Japan

**Keywords:** East Asia, Southeast Asia, biogeography, classification, trapdoor spiders, living fossils

## Abstract

The spider suborder Mesothelae, containing a single extant family Liphistiidae, represents a species-poor and ancient lineage. These are conspicuous spiders that primitively retain a segmented abdomen and appendage-like spinnerets. While their classification history is nearly devoid of phylogenetic hypotheses, we here revise liphistiid genus level taxonomy based on original sampling throughout their Asian range, and on the evidence from a novel molecular phylogeny. By combining morphological and natural history evidence with phylogenetic relationships in the companion paper, we provide strong support for the monophyly of Liphistiidae, and the two subfamilies Liphistiinae and Heptathelinae. While the former only contains *Liphistius* Schiödte, 1849, a genus distributed in Indonesia (Sumatra), Laos, Malaysia, Myanmar, Thailand, we recognize and diagnose seven heptatheline genera, all but three removed from the synonymy of *Heptathela*: i) *Ganthela* Xu & Kuntner, **gen. n.** with the type species *Ganthela yundingensis* Xu, **sp. n.** is known from Fujian and Jiangxi, China; ii) a rediagnosed *Heptathela* Kishida, 1923 is confined to the Japanese islands (Kyushu and Okinawa); iii) *Qiongthela* Xu & Kuntner, **gen. n.** with the type species *Qiongthela baishensis* Xu, **sp. n.** is distributed disjunctly in Hainan, China and Vietnam; iv) *Ryuthela* Haupt, 1983 is confined to the Ryukyu archipelago (Japan); v) *Sinothela* Haupt, 2003 inhabits Chinese areas north of Yangtze; vi) *Songthela* Ono, 2000 inhabits southwest China and northern Vietnam; and vii) *Vinathela* Ono, 2000 (*Abcathela* Ono, 2000, **syn. n.**; *Nanthela* Haupt, 2003, **syn. n.**) is known from southeast China and Vietnam.

## Introduction

The only extant family within the spider suborder Mesothelae, the family Liphistiidae consists of only 88 extant species-level taxa currently grouped in three genera, and displays an interesting geographical distribution confined to Southeast and East Asia (World Spider Catalog 2015). Liphistiids are relatively large, extremely long-lived (5–18 years), ground-dwelling spiders that build trapdoor burrows used for prey capture, shelter and protection (Bristowe 1976, Coddington and Levi 1991, Haupt 2003a). Despite being large and morphologically distinct, they are rarely encountered, making it difficult to secure taxonomically meaningful samples. Their natural history also suggests that liphistiids are confined to their burrows and that the spiders rarely move around, and phylogenetic and biogeographic analyses confirm that they are dispersal-limited and highly genetically structured (Xu et al. submitted).

Since their discovery (Schiödte 1849), much attention has been paid to taxonomy, and most authors (e.g. Schwendinger and Ono 2011) divide Liphistiidae into two distinct subfamilies, Liphistiinae Thorell, 1869 and Heptathelinae Kishida, 1923. Liphistiinae contains a single genus, *Liphistius* Schiödte, 1849 with 50 species-level taxa. Their genital morphology is quite distinct from the 38 currently known species of Heptathelinae, the latter including two currently valid genera, *Heptathela* Kishida, 1923 and *Ryuthela* Haupt, 1983 (World Spider Catalog 2015). Liphistiines are also geographically separated from heptathelines, since *Liphistius* occurs in Southeast Asia (Indonesia (Sumatra), Laos, Malaysia, Myanmar, Thailand), whereas the heptathelines *Heptathela* and *Ryuthela* are confined to East Asia (China, Japan and Vietnam), and Japanese Ryukyu Islands, respectively (World Spider Catalog 2015). Non-taxonomic studies of these spiders have focused on genital evolution (Osaki 1969, Kraus 1978, 1984, Haupt 1983, Yin et al. 1983, 1988, Yin 2001), life history (Yoshikura 1954, 1955, Haupt 1979, 1983, 1984, 1986, 1991, 2003a, Platnick and Sedgwick 1984, Schwendinger 1990), prey-capture (Haupt 1979, 1992, 2003a, Chen et al. 1981, Klingel 1967), mating behaviour (Murphy and Platnick 1981; Haupt 1977, 1979, 1983, 1984, 1992, 2003a, Haupt and Traue 1986, Schwendinger 1990), ecology (Murakami 1934, Klingel 1967, Bristowe 1976, Kikuya 1980, 1982, 1994, Schwendinger 1987, 1988, 1990, 1993, Schwendinger and Pape 2000, Haupt 2003a), zoogeography (Paik 1953, Ono 2000, Haupt 2003a, 2003b) and silk biology (Marples 1967, Haupt 1979, 1983, 1991, 1992, 2003a, Küchler 1987, Haupt and Kovoor 1993, Craig 1997, 2003, Vollrath and Selden 2007, Swanson et al. 2009, Strarrett et al. 2012).

All existing classification schemes for Mesothelae and Liphistiidae were dominated by a few selected characters and opinion rather than phylogenetic analyses. Schiödte (1849) described the first species of the genus *Liphistius* (*Liphistius desultor*) and Thorell (1869) placed it in Liphistioidae (sic). Simon (1903) nominated a new genus *Anadiasthothela*, but the species *Anadiastothele thorelli* was a synonym of *Liphistius sumatranus* Thorell, 1890 (see Bristowe 1932). Kishida (1923) erected a new genus *Heptathela* (based on *Liphistius kimurai* Kishida, 1920), and divided the family Liphistiidae into two subfamilies, Liphistiinae (including the tribes Liphistiiae and Heptatheleae) and Anadiasthothelinae (*Anadiasthothele* Simon, 1903) based on details on spinnerets. In 1939, Petrunkevitch raised *Heptathela* to the family rank (Heptathelidae) to include Japanese and Chinese species. This classification system was retained until Haupt (1983, 1990) proposed dividing the group into three genera and two families (Liphistiidae (*Liphistius*), Heptathelidae (*Heptathela* and *Ryuthela*)). Ono’s (2000) scheme treated the groups as two subfamilies: Liphistiinae and Heptathelinae. To *Heptathela* and *Ryuthela*, Ono (2000) added three new heptatheline genera, *Abcathela*, *Songthela* and *Vinathela*, solely based on the female genital morphology. However, Haupt (2003a) continued to prefer his two-family system (Haupt 1984, 1990), rejected *Abcathela* and *Vinathela*, considered *Songthela* as a synonym of *Sinothela* Haupt, 2003, and erected *Nanthela* Haupt, 2003. In the most recent classification scheme of the family, Schwendinger and Ono (2011) rejected all but three genera: *Liphistius* (Liphistiinae), *Heptathela* (Heptathelinae) and *Ryuthela* (Heptathelinae). However, they expressed some doubt at the validity of the genus *Heptathela*, with no fewer than 33 nominal taxa. According to these authors, *Heptathela* may need to be split again if a comprehensive revision and/or phylogeny was to suggest this.

A modern, species-level phylogeny of liphistiid spiders necessary for addressing taxonomic, evolutionary, and biogeographic questions has been long overdue. In a sister paper (Xu et al. submitted), we used molecular data from our original extensive sampling to test the monophyly of the family Liphistiidae and the genera within. Based on a species-level multi-locus phylogeny reported in that paper, and on morphological and natural history diagnostic characters provided here, we revise below the higher level systematics of the family.

## Materials and methods

**Figure 1.**
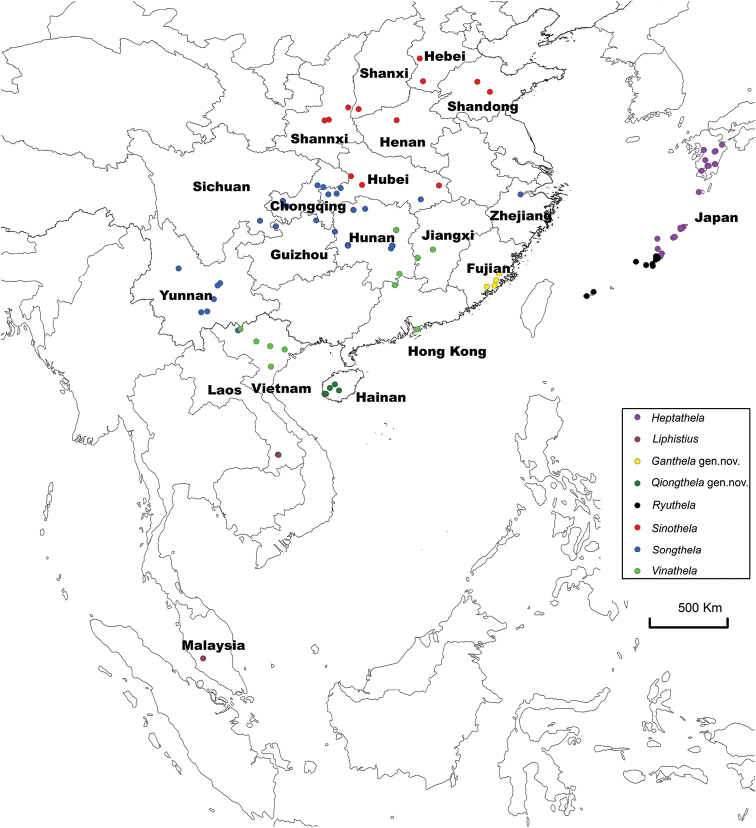
Map showing the sampling localities of liphistiid spider specimens across Southeast and East Asia.

In order to secure a comparative sample of these seemingly rare spiders, we sampled liphistiids through China, Japan and Vietnam both at type locations and in areas with suitable habitat. We collected adults and immature spiders by excavating them from their subterranean burrows, then reared juveniles to adulthood in the laboratory. Since we primarily focused on heptathelines (the liphistiids of East Asia), our sample is biased toward China, Japan, and Vietnam (Figure 1).

Specimens were studied using an Olympus SZX16 stereomicroscope, and anatomical details were examined and photographed with Leica M205C stereomicroscope and Olympus BX51 compound microscope. Genitalia were cleared in boiling KOH for a few minutes to dissolve soft tissues. Unless otherwise noted left palps were depicted. All measurements are in millimeters. Leg and palp measurements are given in the following order: total length (femur + patella + tibia + metatarsus + tarsus).

Abbreviations used are: ALE = anterior lateral eyes, AME = anterior median eyes, BK = book lung, BL = body length, CL = carapace length, Co = conductor, CT = contrategulum, CW = carapace width, D = depression, E = embolus, OL = opisthosoma length, OW = opisthosoma width, PC = paracymbium, PLE = posterior lateral eyes, PME = posterior median eyes, PP = poreplate, RC = receptacular cluster, S = spinneret, SE = sternite, ST = sternum, T = tegulum, TG = tergite, TiA = tibial apophysis.

## Results

In three years we accumulated 1,455 specimens (786 females, 118 males and 551 juveniles) from 145 localities in China, Japan, Laos, Malaysia and Vietnam. These vouchers, deposited at the Centre for Behavioural Ecology and Evolution (CBEE), College of Life Sciences, Hubei University, Wuhan, China, were the basis for our morphological examinations (reported here) and for molecular analyses (Xu et al. submitted). Examined and illustrated specimens were labelled with unique codes (Appendix 1; see also Figure legends), which will be reused in the upcoming genus-level revisions. All designated type specimens were deposited at the National Zoological Museum of China (NZMC), Institute of Zoology, Chinese Academy of Sciences, Beijing, China.

Our trips to chosen sampling points based on the known records were highly successful, and we found heptathelines at most type localities except for *Ryuthela iheyana* from Ihayajima, Japan, *Sinothela sinensis* (Bishop & Crosby, 1932), comb. n. from the type locality, Jinan City, Shandong Province, *Sinothela schensiensis* (Schenkel, 1953), comb. n. from Tongyuan County, Shannxi Province, *Songthela hunanensis* (Song & Haupt, 1984), comb. n. from Qianyang County, Hunan Province, *Songthela yunnanensis* (Song & Haupt, 1984), comb. n. from Kunming, Yunnan Province. We did not sample *Qiongthela nui* (Schwendinger & Ono, 2011), comb. n. and *Qiongthela australis* (Ono, 2002), comb. n. from Lam Dong Province. Most of the field expeditions into previously unsampled areas in China were also successful. New liphistiid localities include Chongqing, Fujian (Putian, Quanzhou and Xiamen), Guizhou (Chishui and Yanhe), Hainan, Hebei (Yongnian), Hubei (Badong, Enshi, Jianshi, Lichuan and Yichang), Jiangxi (Ji’an), Yunan (Dali, Kunming, Mojiang and Yuanjian), and Shandong (Zhangqiu and Yiyuan) Provinces.

**Figure 2.**
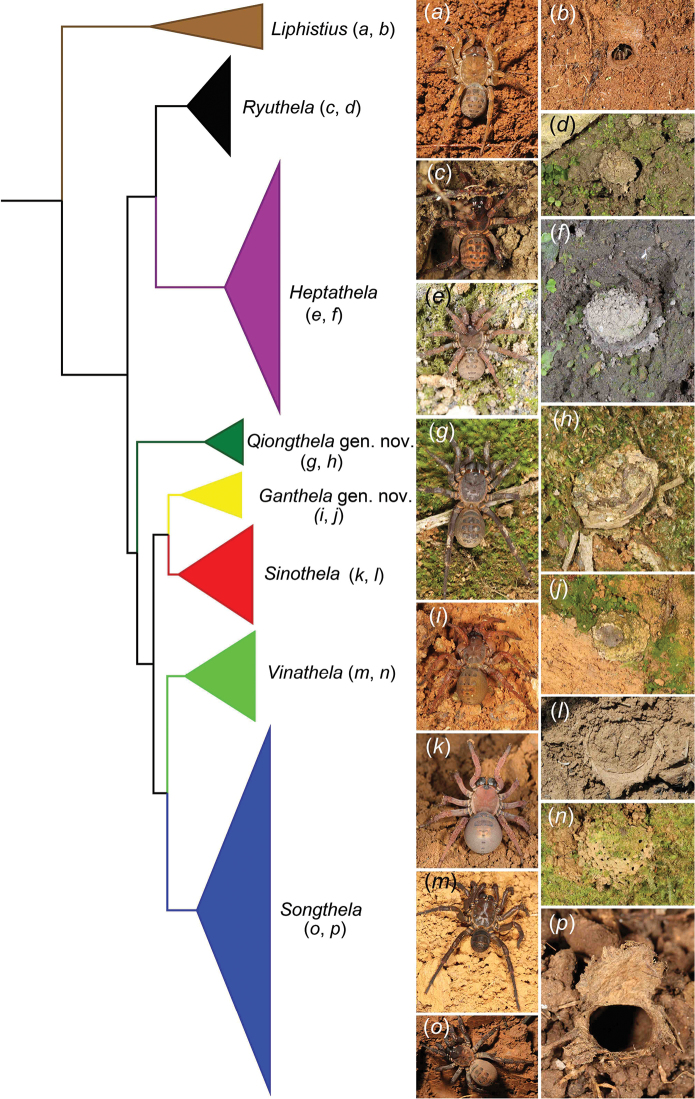
A simplified genus level phylogeny derived from the summary tree in the accompanying paper (Xu et al. submitted), as the basis for newly proposed classification. Images on the right depict typical generic characteristics (female habitus and trapdoor).

In a concurrent paper (Xu et al. submitted), we report on phylogenetic analyses using original five-gene nucleotide data for 75 species. These results, summarized in Figure 2, form the phylogenetic basis for a revised classification of the family. The family and subfamily monophyly were well supported in all phylogenetic analyses (for details, see Xu et al. submitted). The current classification of Liphistiidae, based on morphological features, treats as valid three genera (see World Spider Catalog 2015): *Heptathela* s.l., *Liphistius* and *Ryuthela*. Our phylogenetic results strongly support the monophyly of *Liphistius* and *Ryuthela*, but not of *Heptathela* s.l., and thus require substantial taxonomic emendations. Below, we classify the species currently in paraphyletic *Heptathela* s.l. in six genera—the monophyly of each strongly supported (for details, see Xu et al. submitted)—of which two are new, describe two new species that become the type for the new genera, and propose further synonymies.

## Taxonomy

### Suborder Mesothelae

Taxon classificationAnimaliaAraneaeLiphistiidae

Pocock, 1892

#### Phylogenetic definition

**(for details, see Xu et al. submitted).** In the analysis of divergence times, we treated Mesothelae as a stem group leading from the root of all spiders to the node-based clade Liphistiidae. Therefore, Mesothelae accommodates the fossil genus *Palaeothele* Selden, 2000, which does not share one of the synapomorphies of Liphistiidae (single row of teeth on cheliceral fang groove). Although the morphological diagnosis resembles that of Liphistiidae, Mesothelae is inclusive of Liphistiidae but the two groups are phylogenetically not identical.

#### Composition.

Mesothelae includes the crown group Liphistiidae with extant species from East and Southeast Asia, and the fossil *Palaeothele montceauensis* (Selden 1996a, b) from the Upper Carboniferous of Montceau-les-Mines, France around 295 Ma.

### Family Liphistiidae

Taxon classificationAnimaliaAraneaeLiphistiidae

Thorell, 1869

#### Diagnosis.

**Figures 3–4.**
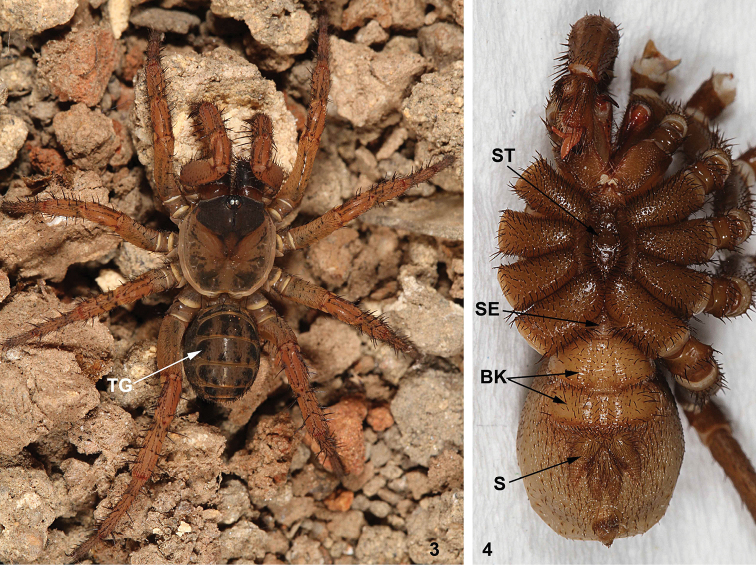
General somatic morphology of Liphistiidae. **3** Female *Heptathela yanbaruensis* Haupt, 1983 (XUX-2014–038A) **4** Male *Qiongthela baishensis* sp. n. (XUX-2012–087). BK = book lung, S = spinneret, SE = sternite, ST = sternum, TG = tergite.

Unlike all other extant spiders, Liphistiidae possess tergites on all abdominal segments (Figure 3), their spinnerets are located in the middle of abdominal venter (Figure 4), and in addition to a narrow sternum they also possess another narrow ventral plate, the sternite, located adjacent to coxae IV (Figure 4).

#### Description.

Medium to large sized ground dwelling and burrowing spiders, chelicerae with a single row of teeth, two pairs of book lungs (Figure 4), tibial spurs specialized as sense organs. Their ground burrows are closed with trapdoors, with or without additional concentric signal lines (Figure 2b, d, f, h, j, l, n, p).

**Composition.** *Ganthela* Xu & Kuntner, gen. n., *Heptathela* Kishida, 1923, *Liphistius* Schiödte, 1849, *Qiongthela* Xu & Kuntner, gen. n., *Ryuthela* Haupt, 1983, *Sinothela* Haupt, 2003a, *Songthela* Ono, 2000, and *Vinathela* Ono, 2000.

#### Distribution.

China, Indonesia (Sumatra), Japan, Laos, Malaysia, Myanmar, Thailand and Vietnam.

### Subfamily Liphistiinae

Taxon classificationAnimaliaAraneaeLiphistiidae

Thorell, 1869

#### Diagnosis.

**Figures 5–9.**
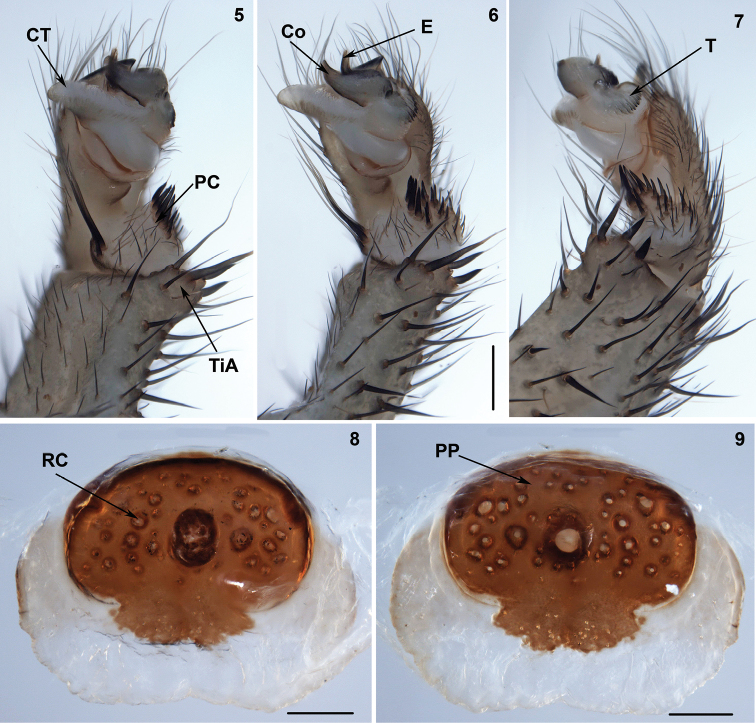
Male (XUX-2013–267) and female (XUX-2013–256) genital anatomy of *Liphistius laoticus* Schwendinger, 2013. **5** palp prolateral view **6** palp ventral view **7** palp retrolateral view **8** vulva ventral view **9** vulva dorsal view. Scales **3–5:** 0.5 mm, **6–7:** 0.1 mm. Co = conductor, CT = contrategulum, E = embolus, PC = paracymbium, PP = poreplate, RC = receptacular cluster, T = tegulum, TiA = tibial apophysis.

In contrast to the members of the subfamily Heptathelinae, Liphistiinae spiders construct signal lines radiating from the burrow entrance (Figure 2b), the male palp possesses a tibial apophysis (Figures 5–7), and the female genitals have a poreplate and unpaired receptacular clusters (Figures 8–9). Platnick and Sedgwick (1984) also report the unique presence of clavate trichobothria on the tarsi and metatarsi of all legs and on the palpal tarsi.

#### Composition.

*Liphistius* Schiödte, 1849.

#### Distribution.

Indonesia (Sumatra), Laos, Malaysia, Myanmar, Thailand.

### Genus Liphistius

Taxon classificationAnimaliaAraneaeLiphistiidae

Schiödte, 1849

Figures 5–9

Liphistius Schiödte, 1849, type species *Liphistius desultor* Schiödte, 1849, P. 621.
Anadiastothele Simon, 1903, type species by original designation *Anadiastothele thorelli* Simon, 1903 = *Liphistius sumatranus* Thorell, 1890, P. 875; first synonymised by Bristowe, 1932, P. 1022.

#### Diagnosis.

See Liphistiinae.

#### Description.

Total length (excluding chelicerae) = 9–37 mm (Platnick and Sedgwick 1984); male palp with retrolateral tibial apophysis bearing strong apical spines and with a spinose paracymbium; female genitalia with a poreplate and unpaired receptacular clusters.

#### Species composition.

*Liphistius albipes* Schwendinger, 1995; *Liphistius batuensis* Abraham, 1923; *Liphistius bicoloripes* Ono, 1988; *Liphistius birmanicus* Thorell, 1897; *Liphistius bristowei* Platnick & Sedgwick, 1984; *Liphistius castaneus* Schwendinger, 1995; *Liphistius dangrek* Schwendinger, 1996; *Liphistius desultor* Schiödte, 1849; *Liphistius endau* Sedgwick & Platnick, 1987; *Liphistius erawan* Schwendinger, 1996; *Liphistius fuscus* Schwendinger, 1995; *Liphistius isan* Schwendinger, 1998; *Liphistius jarujini* Ono, 1988; *Liphistius johore* Platnick & Sedgwick, 1984; *Liphistius kanthan* Platnick, 1997; *Liphistius lahu* Schwendinger, 1998; *Liphistius langkawi* Platnick & Sedgwick, 1984; *Liphistius lannaianus* Schwendinger, 1990; *Liphistius laoticus* Schwendinger, 2013; *Liphistius laruticus* Schwendinger, 1997; *Liphistius lordae* Platnick & Sedgwick, 1984; *Liphistius malayanus* Abraham, 1923; *Liphistius malayanus cameroni* Haupt, 1983; *Liphistius marginatus* Schwendinger, 1990; *Liphistius murphyorum* Platnick & Sedgwick, 1984; *Liphistius nesioticus* Schwendinger, 1996; *Liphistius niphanae* Ono, 1988; *Liphistius ochraceus* Ono & Schwendinger, 1990; *Liphistius onoi* Schwendinger, 1996; *Liphistius ornatus* Ono & Schwendinger, 1990; *Liphistius owadai* Ono & Schwendinger, 1990; *Liphistius panching* Platnick & Sedgwick, 1984; *Liphistius phileion* Schwendinger, 1998; *Liphistius phuketensis* Schwendinger, 1998; *Liphistius pusohm* Schwendinger, 1996; *Liphistius rufipes* Schwendinger, 1995; *Liphistius sayam* Schwendinger, 1998; *Liphistius schwendingeri* Ono, 1988; *Liphistius sumatranus* Thorell, 1890; *Liphistius suwat* Schwendinger, 1996; *Liphistius tempurung* Platnick, 1997; *Liphistius tenuis* Schwendinger, 1996; *Liphistius thaleban* Schwendinger, 1990; *Liphistius thaleri* Schwendinger, 2009; *Liphistius tham* Sedgwick & Schwendinger, 1990; *Liphistius thoranie* Schwendinger, 1996; *Liphistius tioman* Platnick & Sedgwick, 1984; *Liphistius trang* Platnick & Sedgwick, 1984; *Liphistius yamasakii* Ono, 1988; *Liphistius yangae* Platnick & Sedgwick, 1984.

#### Distribution.

Indonesia (Sumatra), Laos, Malaysia, Myanmar, Thailand.

#### Remarks.

*Liphistius* always possess eight spinnerets, unlike all the remaining liphistiid genera in which the number is variable (either seven or eight). Therefore, the number of spinnerets is not a criterion for discriminating genera and species (Haupt 1983).

### Subfamily Heptathelinae

Taxon classificationAnimaliaAraneaeLiphistiidae

Kishida, 1923

#### Diagnosis.

In contrast to the members of the subfamily Liphistiinae, the representatives of Heptathelinae lack signal lines radiating from the burrow entrance (Figure 2d, f, h, j, l, n, p), the male palp lacks a tibial apophysis (Figures 10–12, 15–17, 20–22, 26–28, 31–33, 36–38, 41–43), and the female genitals have paired or unpaired receptacular clusters on the bursa copulatrix with or without stalks (Figures 13–14, 18–19, 25, 34–35, 39–40, 44–45).

**Figures 10–14.**
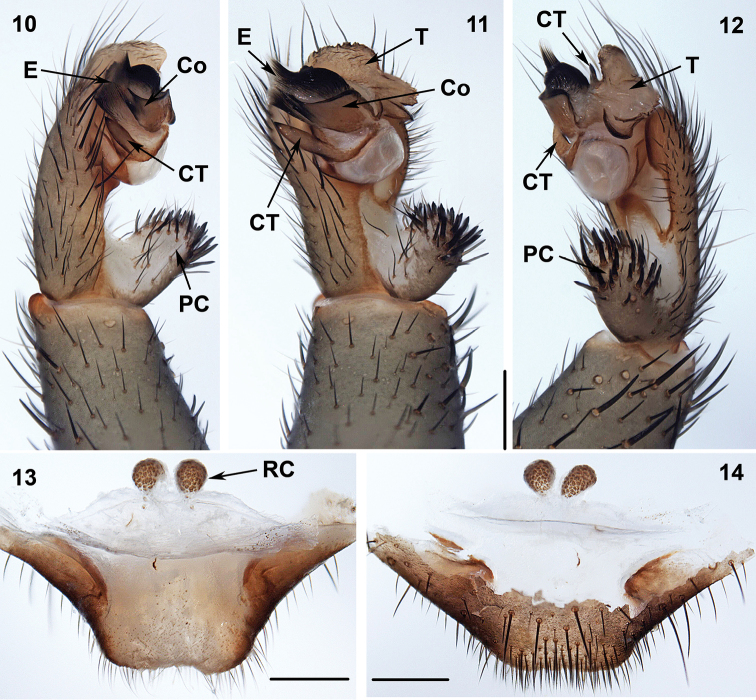
Male (XUX-2013–136) and female (XUX-2013–135) genital anatomy of *Ganthela yundingensis* sp. n. **10** palp prolateral view **11** palp ventral view **12** palp retrolateral view **13** vulva dorsal view **14** vulva ventral view. Scales 0.5 mm. RC = receptacular cluster.

**Figures 15–21.**
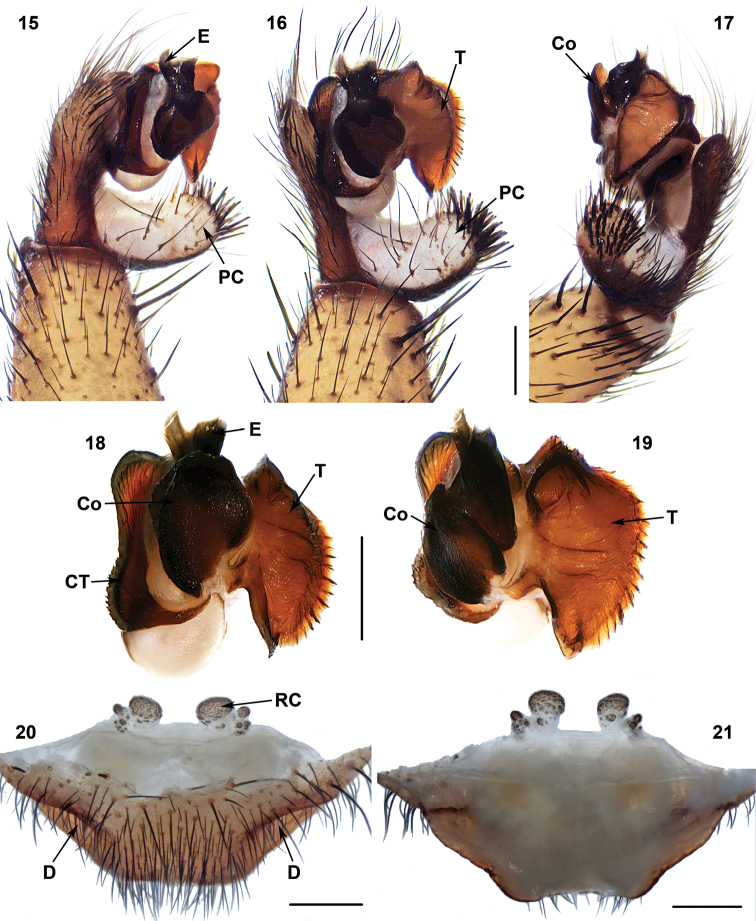
Male (XUX-2013–389) and female (XUX-2013–351) genital anatomy of *Heptathela higoensis* Haupt, 1983 and *Heptathela kimurai* (Kishida, 1920), respectively. **15** palp prolateral view **16** palp ventral view **17** palp retrolateral view **18** contrategulum, conductor and embolus, ventral view **19** contrategulum, conductor and embolus, retrolateral view **20** vulva ventral view **21** vulva dorsal view; Scales 0.5 mm. D = depression.

**Figures 22–27.**
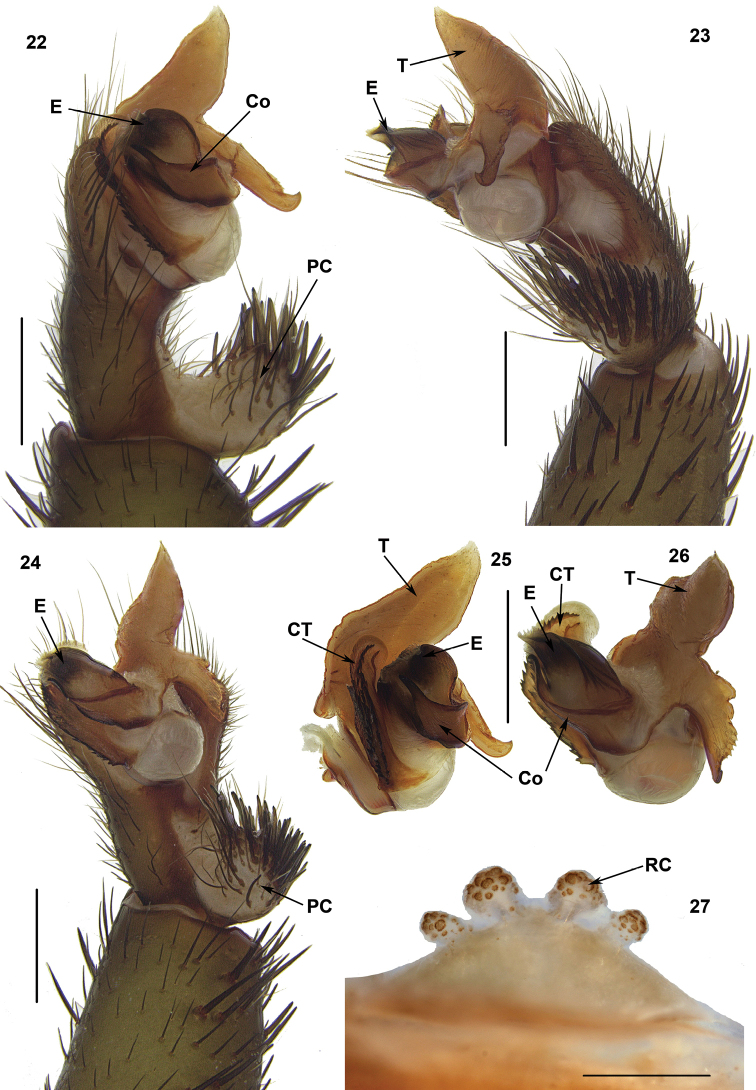
Male (XUX-2012–087) and female (XUX-2012–086) genital anatomy of *Qiongthela baishensis* sp. n. **22** palp prolateral view **23** palp retrolateral view **24** palp ventral view **25–26** contrategulum, conductor and embolus, distal view **27** vulva dorsal view. Scales **18–20:** 1 mm, **21–23:** 0.5 mm.

**Figures 28–34.**
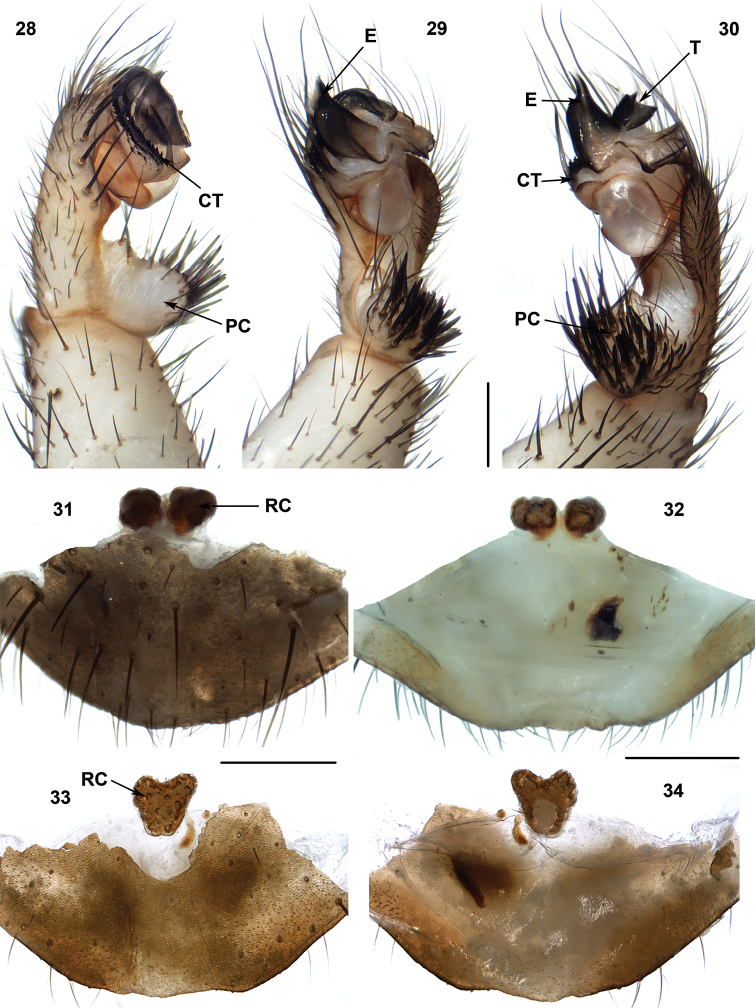
Male (XUX-2013–228) and female (**31–32** XUX-2012–302 and **33–34** XUX-2012–364) genital anatomy of *Ryuthela ishigakiensis* Haupt, 1983, *Ryuthela nishihirai* (Haupt, 1979), and *Ryuthela sasakii* Ono, 1997, respectively. **28** palp prolateral view **29** palp ventral view **30** palp retrolateral view **31, 33** vulva ventral view **32, 34** vulva dorsal view. Scales 0.5 mm.

**Figures 35–39.**
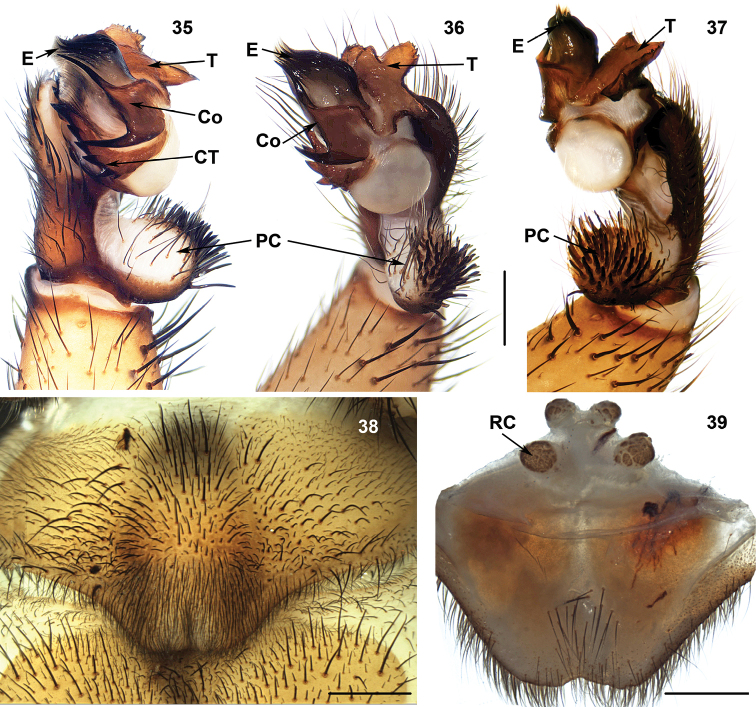
Male (XUX-2012–045) and female (XUX-2012–035) genital anatomy of *Sinothela sinensis* (Bishop & Crosby, 1932), comb. n. **35** palp prolateral view **36** palp ventral view **37** palp retrolateral view **38** vulva ventral view **39** vulva dorsal view. Scales 0.5 mm.

**Figures 40–44.**
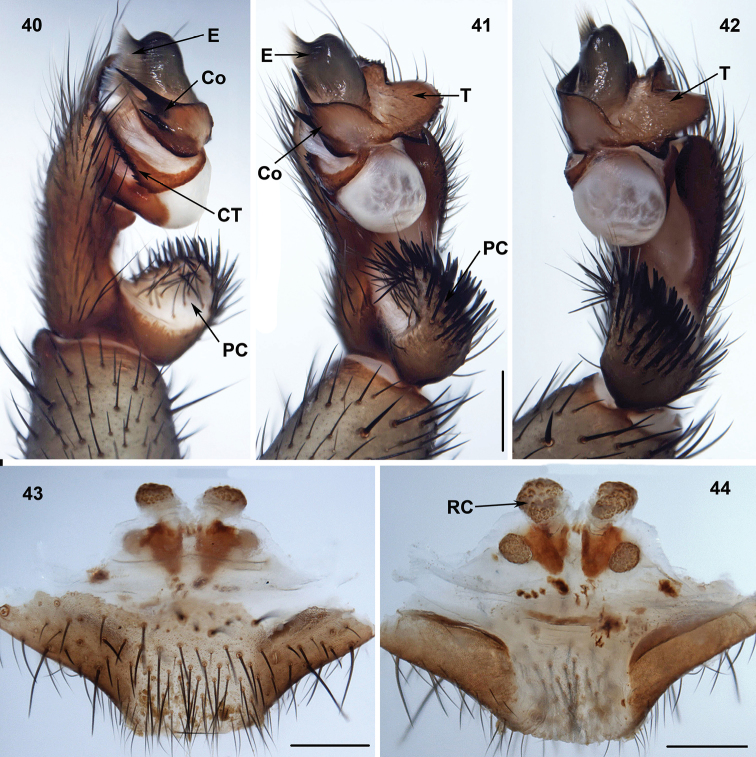
Male (XUX-2013–175) and female (XUX-2013–170) genital anatomy of *Songthela hangzhouensis* (Chen, Zhang & Zhu, 1981), comb. n. **40** palp prolateral view **41** palp ventral view **42** palp retrolateral view **43** vulva ventral view **44** vulva dorsal view. Scales 0.5 mm.

**Figures 45–48.**
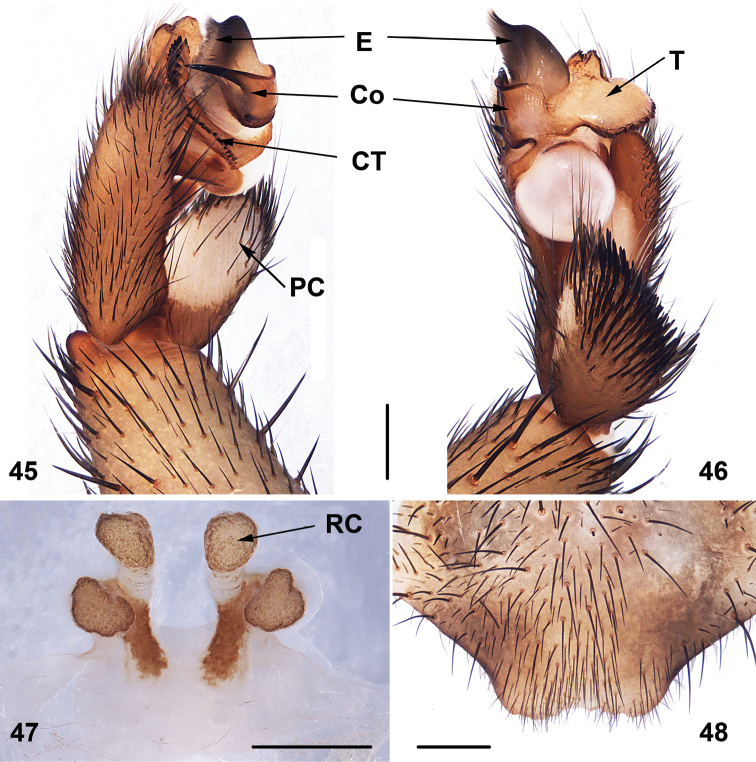
Male (XUX-2011–078) and female (XUX-2011–043) genital anatomy of *Songthela goulouensis* (Yin, 2001), comb. n. **45** palp prolateral view **46** palp retrolateral view **47** vulva ventral view **48** vulva dorsal view. Scales 0.5 mm.

#### Composition.

*Ganthela* Xu & Kuntner, gen. n., *Heptathela* Kishida, 1923, *Qiongthela* Xu & Kuntner, gen. n., *Ryuthela* Haupt, 1983, *Sinothela* Haupt, 2003a, *Songthela* Ono, 2000, and *Vinathela* Ono, 2000.

#### Distribution.

China, Japan and Vietnam.

### Genus Ganthela

Taxon classificationAnimaliaAraneaeLiphistiidae

Xu & Kuntner gen. n.

http://zoobank.org/F70E24D5-C13B-4195-825C-A69A684AB893

Figures 10–14

Liphistius : Wang 1989, P. 30, description of *Liphistius cipingensis* (= *Ganthela cipingensis*).
Songthela : Ono 2000, P. 150, transferred *Liphistius cipingensis* to *Songthela cipingensis* (= *Ganthela cipingensis*).
Heptathela : Platnick 1993, P. 77, transferred *Liphistius cipingensis* to *Heptathela cipingensis* (= *Ganthela cipengensis*).

#### Type species.

*Ganthela yundingensis* sp. n.

#### Etymology.

The genera of heptathelines contain in their name the word ‘thela’ referring to spinnerets as the Greek word *thele* means nipple-like protuberance (Ono 2000). We continue this tradition, but name the genus to start with Gan-, which refers to Jiangxi Province.

#### Diagnosis.

Males of *Ganthela* differs from all other Heptathelinae genera by a smooth conductor with a distal spiniform apex (Figures 10–11), a flat opening embolus and scale-like contrategulum (Figures 10–11), females can be identified by a single pair of similar receptacular clusters (Figures 13–14).

#### Description.

Total length (excluding chelicerae) = 8–15 mm (N = 35); male palpal conductor smooth, wide, leaf-shaped, with a spiniform apex (Figures 10–11); spinose paracymbium relative short (Figures 10, 12); embolus with a flat opening (Figures 10–11); contrategulum scale-like with a smooth margin (Figures 10–11); female genitalia with paired receptacular clusters of similar size, situated at the anterior margin of the bursa copulatrix with tubular stems (Figures 13–14).

#### Species composition.

*Ganthela cipingensis* (Wang, 1989), comb. n. (7♀), male is unknown, *Ganthela yundingensis* Xu, sp. n. (1♂1♀), one undescribed species from Jiangxi Province, China (11♀), and four undescribed species from Fujian Province, China (1♀, 1♂11♀, 1♀, and 3♀, respectively).

#### Distribution.

China (Fujian, Jiangxi).

#### Remarks.

Wang (1989) placed *Ganthela cipingensis* in *Liphistius* based on the presence of eight spinnerets. Our collections from the type locality contain specimens with seven spinnerets. The number of spinnerets thus varies intraspecifically.

### Ganthela yundingensis

Taxon classificationAnimaliaAraneaeLiphistiidae

Xu sp. n.

http://zoobank.org/4F54A057-613A-4E8E-ADB4-B1B0AA0BB579

Figures 10–14

#### Types.

Male holotype (XUX-2013–136) and female paratype from Mt. Yunding, Tingxi Town, Tong’an District, Xiamen City, Fujian Province, China; 24.87°N, 118.16°E, 631 m; 8 July 2013; collected by F. Liu, X. Xu and Z. Zhang, deposited at NZMC, Institute of Zoology, Chinese Academy of Sciences, Beijing, China.

#### Etymology.

‘Yunding’ refers to the type locality of this species, Mt. Yunding.

#### Diagnosis.

Females can be distinguished from *Ganthela cipingensis* and the five undescribed *Ganthela* species we are aware of by lacking genital stalks (Figures 13–14), and the males, uniquely among heptathelines, possess the contrategulum with two marginal apophyses (Figures 11–12), the prolateral one being scale-like (Figure 11).

#### Description.

Male (holotype). Carapace and opisthosoma light brown; tergites dark brown; sternum narrow, twice as long as wide; a few long pointed hairs running over ocular mound in a longitudinal row; chelicerae robust with promargin of cheliceral groove with 10 denticles of variable size; legs with strong hairs and spines; opisthosoma with 12 tergites, the first 2–5 larger than others and the fourth largest, the first four close to each other; 7 spinnerets. Measurements: BL 9.80, CL 4.48, CW 4.03, OL 4.98, OW 3.75; ALE > PLE > PME > AME; leg I 13.60 (4.03 + 1.48 + 2.90 + 3.41 + 1.78), leg II 13.80 (3.81 + 1.70 + 2.91 + 3.48 + 1.90), leg III 16.01 (4.02 + 1.71 + 3.28 + 4.58 + 2.42), leg IV 20.60 (5.20 + 1.89 + 3.90 + 6.50 + 3.11).

Palp: Cymbium with a projection; prolateral side of paracymbium unpigmented and unsclerotised, numerous setae and spines at the tip of paracymbium (Figures 10–12). Contrategulum has two marginal apophyses, the first one scale-like with a smooth margin (Figures 10–11). Tegulum with a dentate edge (Figures 11–12). Conductor wide leaf-shaped, with spiniform apex, parallel to embolus (Figures 10–11). Embolus largely sclerotized, with a flat opening (Figures 10–11).

Female. Colouration of carapace and opisthosoma as in male; chelicerae robust with promargin of cheliceral groove with 12 strong denticles of variable size; legs with strong hairs and spines; opisthosoma with 12 tergites, as in male; 7 spinnerets. Measurements: BL 13.23, CL 5.96, CW 5.18, OL 7.28, OW 4.90; ALE > PLE > PME > AME; palp 9.64 (3.26 + 1.61 + 2.15 + 2.62), leg I 11.46 (3.33 + 2.08 + 2.17 + 2.30 + 1.58), leg II 11.82 (3.56 + 2.11 + 2.13 + 2.42 + 1.60), leg III 13.18 (3.71 + 2.20 + 2.33 + 3.02 + 1.92), leg IV 17.59 (4.03 + 2.40 + 3.30 + 5.19 + 2.67).

Female genitalia: The posterior part of the genital area rectangular (Figure 13–14), a pair of receptacular clusters close to each other, without stalks (Figures 13–14).

### Genus Heptathela

Taxon classificationAnimaliaAraneaeLiphistiidae

Kishida, 1923

Figures 15–21

Heptathela Kishida, 1923, type species *Liphistius kimurai* Kishida, 1920, P. 235.

#### Diagnosis.

*Heptathela* males differ from all other Heptathelinae genera by a leaf-shaped conductor (Figures 18–19), a thumb-shaped embolus (Figures 15, 18) and a wide tegulum with a rugate margin (Figures 16, 18–19). *Heptathela* females can be distinguished from all other Heptathelinae genera by a single paired depression on the ventro-lateral part of genital atrium (Figure 20), and by the one pair of main receptacular cluster and secondary, lateral, irregular receptacular clusters (Figures 20–21).

#### Description.

Total length (excluding chelicerae) = 7–17 mm (N = 229); male palp with a leaf-shaped conductor with spiniform apex or dentate edge, rugate (Figures 18–19); spinose paracymbium long, nearly the length of the cymbium (Figures 15–16); embolus thumb-shape (Figures 15, 18); tegulum wide, with a rugate margin (Figures 16, 18–19); female genitalia with a paired depression on the ventro-lateral part of the genital atrium (Figure 20); with a pair of main receptacular cluster at the anterior margin of the bursa copulatrix and separated from each other, and with secondary, lateral, irregular receptacular clusters (Figures 20–21).

#### Species composition.

*Heptathela amamiensis* Haupt, 1983; *Heptathela higoensis* Haupt, 1983; *Heptathela kanenoi* Ono, 1996; *Heptathela kikuyai* Ono, 1998; *Heptathela kimurai* (Kishida, 1920); *Heptathela nishikawai* Ono, 1998; *Heptathela yaginumai* Ono, 1998; *Heptathela yakushimaensis* Ono, 1998; *Heptathela yanbaruensis* Haupt, 1983.

#### Distribution.

Japan (Kyushu and Okinawa).

### Genus Qiongthela

Taxon classificationAnimaliaAraneaeLiphistiidae

Xu & Kuntner gen. n.

http://zoobank.org/BA103085-F5FB-4DF1-81C7-CA5CDE26E65E

Figures 22–27

#### Type species.

*Qiongthela baishensis* sp. n.

#### Etymology.

The genera of heptathelines contain in their name the word ‘thela’ referring to spinnerets as the Greek word *thele* means nipple-like protuberance (Ono 2000). We continue this tradition, but name the genus to start with Qiong-, referring to Hainan Province, China.

#### Diagnosis.

*Qiongthela* males differ from all other Heptathelinae genera by the conductor with a narrow, blade-like, slightly hooked apex (Figures 22, 25–26), and by tegulum with two apophyses (Figures 23, 25–26). *Qiongthela* females can be distinguished from all other Heptathelinae by two paired receptacular clusters located at the anterior margin of the bursa copulatrix (Figure 27).

#### Description.

Total length (excluding chelicerae) = 13–31 mm (N = 14); male palp with a distally free conductor, narrow, blade-like with slightly hook-like apex, (Figures 22, 25–26); tegulum with two margins, spinose paracymbium (Figures 23, 25–26); female genitalia with two paired receptacular clusters, all situated at the anterior margin of the bursa copulatrix with more or less distinct tubular stems (Figure 27).

#### Species composition.

*Qiongthela australis* (Ono, 2002), comb. n., *Qiongthela nui* (Schwendinger & Ono, 2011), comb. n., *Qiongthela baishensis* sp. n. (3♂2♀), and three undescribed species (6♂8♀, 1♀ and 1♂1♀, respectively) from Hainan, China.

#### Distribution.

Hainan (China) and Vietnam.

#### Remarks.

Based on morphological descriptions, but not on phylogenetic analyses, we include two species from Vietnam in this genus, originally described as *Songthela australis* Ono, 2002 and *Heptathela nui* Schwendinger & Ono, 2011.

### Qiongthela baishensis

Taxon classificationAnimaliaAraneaeLiphistiidae

Xu sp. n.

http://zoobank.org/5C0F3DB2-3A07-4FC6-83B5-3E286F1493F1

Figures 22–27

#### Types.

Male holotype (XUX-2012–087, matured 10 October 2012 at CBEE, College of Life Sciences, Hubei University) and two male and two female paratypes from Nangaoling Forest Plantation, Baisha County, Hainan Province, China; 19.24°N, 109.38°E, 463 m, collected 18 July 2012 by D. Li, F. Liu and X. Xu, deposited at NZMC, Institute of Zoology, Chinese Academy of Sciences, Beijing, China.

#### Etymology.

The species epithet refers to Baisha, the species type locality.

#### Diagnosis.

Unlike other *Qiongthela* species, males of *Qiongthela baishensis* possess three parallel serrated distal edges of the contrategulum (Figures 25, 26), and females have two pairs of receptacular clusters, the median pair larger than the lateral one, with very short or no stalks (Figure 27).

#### Description.

Male (holotype). Carapace and opisthosoma light brown; tergites darker; with a clear fovea; sternum narrow, nearly twice as long as wide; a few long pointed hairs running over ocular mound in a longitudinal row; chelicerae robust with promargin of cheliceral groove containing 10 denticles of variable size; legs with strong hairs and spines; opisthosoma with 12 tergites, the first 2–7 distinctly larger and the fifth largest; 7 spinnerets. Measurements: BL 16.75, CL 6.70, CW 6.65, OL 9.90, OW 7.45; ALE > PLE > PME > AME; leg I 19.76 (6.15 + 2.55 + 4.35 + 4.35 + 2.36), leg II 20.70 (5.59 + 2.67 + 4.24 + 5.45 + 2.75), leg III 21.16 (5.25 + 2.13 + 4.12 + 6.45 + 3.21), leg IV 26.03 (7.38 + 2.75 + 5.78 + 7.05 + 3.07).

Palp: Cymbium with a projection; prolateral side of paracymbium unpigmented and unsclerotised, numerous setae and spines at the tip of paracymbium (Figures 22, 24). Contrategulum with three parallel distal edges, row of denticles on inner edge running down to ventro-proximal margin of contrategulum and the outer row forming a sharp edge without denticles (Figures 22, 25–26). Tegulum with a very long, wide base, pointed, distally directed marginal apophysis with a sharp edge, and retrolaterally with a proximally directed terminal apophysis with a slightly short dentate row and continuously narrowing to a rounded, hooked apex (Figures 22–26). Conductor situated ventro-proximally on embolus, with a bent apex (Figures 22, 25–26). Embolus largely sclerotised, prolaterally with numerous longitudinal ribs (Figures 22–26).

Female (paratype). Colouration as in male; promargin of robust chelicerae with 9 strong denticles variable in size; legs and opisthosoma as in the male; 7 spinnerets. Measurements: BL 13.30–14.15, CL 4.51–6.23, CW 4.63–5.82, OL 7.20–7.45, OW 4.33–5.08; ALE > PLE > PME > AME; palp 10.25 (3.65 + 1.55 + 2.30 + 2.75), leg I 12.48 (4.25 + 1.95 + 2.53 + 2.55 + 1.20), leg II 12.15 (3.75 + 2.07 + 2.25 + 2.65 + 1.43), leg III 12.42 (3.55 + 2.12 + 2.03 + 3.07 + 1.65), leg IV 19.20 (5.45 + 2.65 + 3.45 + 5.10 + 2.55).

Female genitalia: Two pairs of receptacular clusters along the anterior margin of bursa copulatrix, the median pair larger than the lateral one, with very short or no stalks (Figure 27).

### Genus Ryuthela

Taxon classificationAnimaliaAraneaeLiphistiidae

Haupt, 1983

Figures 28–34

Ryuthela Haupt, 1983, type species *Heptathela nishihirai* Haupt, 1979, P. 286.

#### Diagnosis.

*Ryuthela* males differ from all other Heptathelinae genera by lacking the conductor and by the contrategulum with an enlongate spine (Figures 28, 30). The females differ from *Heptathela*, *Qiongthela*, *Sinothela*, *Songthela* and *Vinathela* by one paired receptacular cluster close to each other (Figures 31–32), located at the anterior margin of the bursa copulatrix, and from *Ganthela* by receptacular clusters without stems that may or may not be fused (Figures 31–34).

#### Description.

Total length (excluding chelicerae) = 7–15 mm (N = 151); male palp with denticulate contrategulum and ventral portion with an elongate spine (Figures 28, 30); spinose paracymbium relatively short (Figures 28–30); female genitalia usually with one paired receptacular clusters, except in some specimens, notably in *Ryuthela sasakii*, where receptacular clusters are unpaired and without stalks (Figures 31–34).

#### Species composition.

*Ryuthela iheyana* Ono, 2002; *Ryuthela ishigakiensis* Haupt, 1983; *Ryuthela nishihirai* (Haupt, 1979); *Ryuthela sasakii* Ono, 1997; *Ryuthela tanikawai* Ono, 1997.

#### Distribution.

Ryukyu Island (Japan).

#### Remarks.

In *Ryuthela*, female genital anatomy shows considerable intraspecific variation, therefore the structure of the male palp appears more reliable for diagnostics and identification.

### Genus Sinothela

Taxon classificationAnimaliaAraneaeLiphistiidae

Haupt, 2003

Figures 35–39

Sinothela Haupt, 2003a, type species *Heptathela sinensis* Bishop & Crosby, 1932; synonymized with *Songthela* by Platnick, 2011; synonymized with *Heptathela* by Schwendinger & Ono, 2011, P. 601. Herein removed from synonymy of *Heptathela*.

#### Diagnosis.

*Sinothela* males differ from all other Heptathelinae genera by the conductor with a smooth surface, its proximal portion being fairly wide, and its distal portion with more than one apical spine (Figures 35–36), and by the contrategulum with large serrations (Figure 35). *Sinothela* females differ from all other Heptathelinae genera by two paired receptacular clusters with the median pair close to each other situated at the basal bursa copulatrix with tubular stem, lateral ones situated on dorsal side (Figure 39).

#### Description.

Total length (excluding chelicerae) = 13–28 mm (N = 71); male palpal conductor smooth, proximally fairly wide, distally with more than one spine tip (Figures 35–36); contrategulum with a serrated edge (Figure 35); tegulum with three apophyses (Figures 35–37); spinose paracymbium relatively short (Figures 35–37); female genitalia with two paired receptacular clusters, median pair close to each other situated at the basal bursa copulatrix with tubular stem, lateral ones situated more dorsally (Figure 39).

#### Species composition.

*Sinothela heyangensis* (Zhu & Wang, 1984), comb. n. (8♂25♀; male previously unknown), *Sinothela luotianensis* (Yin et al., 2002), comb. n. (3♀), *Sinothela schensiensis* (Schenkel, 1953), comb. n., *Sinothela sinensis* (Bishop & Crosby, 1932), comb. n. (2♂9♀).

#### Distribution.

China north of Yangzi River (Hebei, Henan, Hubei, Shandong, Shaanxi, and Shanxi).

### Genus Songthela

Taxon classificationAnimaliaAraneaeLiphistiidae

Ono, 2000

Figures 40–44 , 45–48

Songthela Ono, 2000, type species *Heptathela hangzhouensis* Chen, Zhang & Zhu, 1981; synonymized with *Sinothela* by Haupt, 2003a, P. 71; synonymized with *Heptathela* by Schwendinger & Ono, 2011, P. 601. Herein removed from synonymy of *Heptathela*.

#### Type species.

*Heptathela hangzhouensis* Chen, Zhang & Zhu, 1981.

#### Diagnosis.

*Songthela* males differ from all other heptatheline genera by the conductor with a smooth surface and with the proximal portion relatively narrow, the distal portion with more than one apical spine (Figures 40–41, 45–46), and by the embolus with a flat opening (Figures 40–41, 45–46). *Songthela* females differ from all other heptatheline genera by two paired receptacular clusters, all four of similar size or median ones larger than laterals, median pair with tubular stems situated at the anterior margin of bursa copulatrix, lateral ones situated more dorsally (Figures 43–44, 47).

#### Description.

Total length (excluding chelicerae) = 8–21 mm (N = 304); male palpal conductor with one or two distal spines: the long one nearly reaching the embolus edge, the shorter one positioned at the middle part of conductor (Figures 40–41, 45–46); embolus with a wide, flat opening (Figures 40, 45–46); tegulum with serrated margin (Figures 41–42, 46); spinose paracymbium relatively short (Figures 40–41, 45); female genitalia as diagnosed (Figures 43–44, 47).

#### Species composition.

*Songthela bristowei* (Gertsch, 1967), comb. n. (2♂10♀), *Songthela ciliensis* (Yin, Tang & Xu, 2003), comb. n., *Songthela goulouensis* (Yin, 2001), comb. n. (8♂41♀; male previously unknown), *Songthela hangzhouensis* (Chen, Zhang & Zhu, 1981), comb. n. (4♂10♀), *Songthela jianganensis* (Chen et al., 1988), comb. n. (11♀), *Songthela mangshan* (Bao, Yin & Xu, 2003), comb. n., *Songthela sapana* (Ono, 2010), comb. n. (4♀), *Songthela shei* (Xu & Yin, 2001), comb. n. (3♂4♀; male previously unknown), *Songthela wosanensis* (Wang & Jiao, 1995), comb. n. (2♂7♀; male previously unknown), *Songthela xianningensis* (Yin et al., 2002), comb. n. (1♂23♀; male previously unknown), *Songthela yunnanensis* (Song & Haupt, 1984), comb. n.

#### Distribution.

China (Chongqing, Guizhou, Hubei, Hunan, Jiangxi, Sichuan, Zhejiang, and Yunnan) and northern Vietnam.

### Genus Vinathela

Taxon classificationAnimaliaAraneaeLiphistiidae

Ono, 2000

Figures 49–55

Vinathela Ono, 2000, type species *Heptathela cucphuongensis* Ono, 1999; synonymized with *Heptathela* by Haupt, 2003a: P. 91. Herein removed from synonymy of *Heptathela*.
Abcathela Ono, 2000, type species *Heptathela abca* Ono, 1999, P. 149; placed in the synonymy of *Heptathela* by Haupt, 2003a, P. 71, 79; **syn. n.**
Nanthela Haupt, 2003a, type species *Liphistius tonkinensis* Bristowe in Bristowe and Millot 1933; placed in the synonymy of *Heptathela* by Schwendinger and Ono 2011, P. 601; **syn. n.**

#### Diagnosis.

**Figures 49–55.**
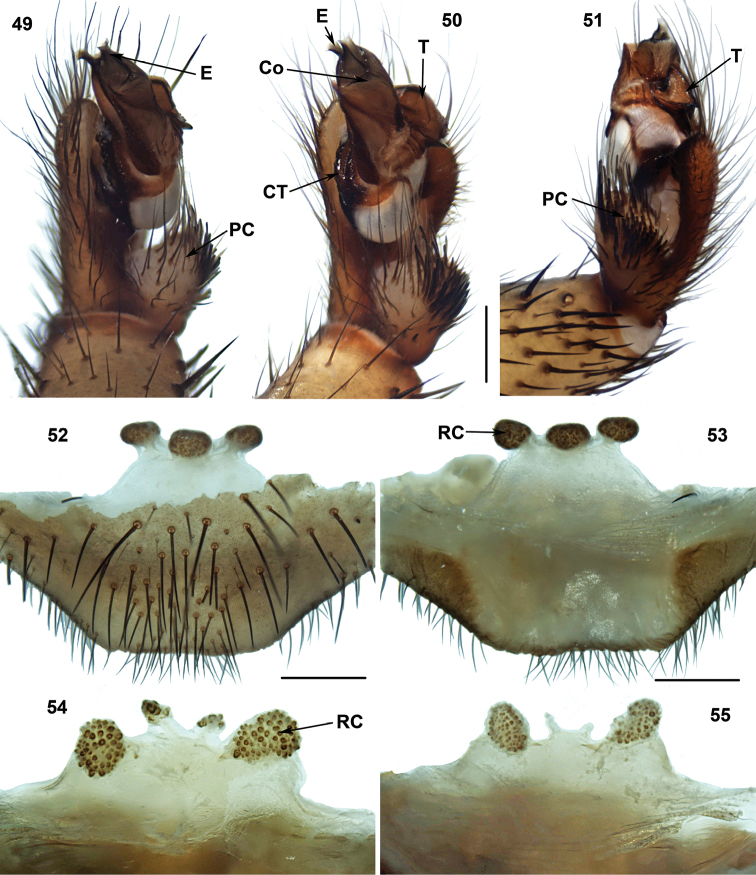
Male (XUX-2013–007) and female genital anatomy of *Vinathela cucphuongensis* (Ono, 1999), comb. n. (52–53: XUX-2013–006) and *Vinathela abca* (Ono, 1999), comb. n. (**54:** XUX-2013–049; **55:** XUX-2013–048) **49** palp prolateral view **50** palp ventral view **51** palp retrolateral view **52** vulva ventral view **53–55** vulva dorsal view. Scales 0.5 mm.

Males of *Vinathela* differ from all other Heptathelinae genera by a wide proximal portion of the conductor, its distal portion being bent (Figure 50), and embolus with two peaks (Figures 49–50); females of *Vinathela* can be distinguished from all other Heptathelinae by three or four receptacular clusters situated at the anterior margin of bursa copulatrix, three of the same size or median pair small and lateral pair large (Figures 52–55).

#### Description.

Total length (excluding chelicerae) = 9–22 mm (N = 71); male palp with long conductor, proximal portion wide, distal portion bent (Figure 50); tegulum thick (Figures 50–51); spinose paracymbium short (Figures 49–51); female genitalia as diagnosed (Figures 52–55).

#### Species composition.

*Vinathela abca* (Ono, 1999), comb. n. (1♂7♀; male previously unknown), *Vinathela cucphuongensis* (Ono, 1999), comb. n. (2♂7♀; male previously unknown), *Vinathela hongkong* (Song & Wu, 1997), comb. n. (3♂19♀; female previously unknown), *Vinathela hunanensis* (Song & Haupt, 1984), comb. n., *Vinathela tomokunii* (Ono, 1997), comb. n. (6♀), *Vinathela tonkinensis* (Bristowe, 1933), comb. n. (1♂4♀; female previously unknown).

#### Distribution.

China (Hong Kong, Hunan and Jiangxi) and Vietnam.

#### Remarks.

*Vinathela* Ono, 2000 has priority over *Nanthela* Haupt, 2003a. We chose *Vinathela* Ono, 2000 over *Abcathela* Ono, 2000 (from the same publication) since the latter also contains species from northern China.

## Supplementary Material

XML Treatment for Mesothelae

XML Treatment for Liphistiidae

XML Treatment for Liphistiinae

XML Treatment for Liphistius

XML Treatment for Heptathelinae

XML Treatment for Ganthela

XML Treatment for Ganthela yundingensis

XML Treatment for Heptathela

XML Treatment for Qiongthela

XML Treatment for Qiongthela baishensis

XML Treatment for Ryuthela

XML Treatment for Sinothela

XML Treatment for Songthela

XML Treatment for Vinathela

## Appendix 1

**Table:** Specimens used in this study. Asterisks mark the previously unknown males.

Species	Specimen code	Sex	Locality	Coordinates	Collectors
*Ganthela yundingensis* sp. n.	XUX-2013-136	male	Mt. Yunding, Tingxi Town, Tong’an District, Xiamen City, Fujian Province, China	24.87827°N; 118.16172°E	Fengxiang Liu, Zengtao Zhang, Xin Xu
	XUX-2013-135	female	Mt. Yunding, Tingxi Town, Tong’an District, Xiamen City, Fujian Province, China	24.87924°N; 118.16194°E	Fengxiang Liu, Zengtao Zhang, Xin Xu
*Heptathela higoensis* Haupt, 1983	XUX-2013-389	male	Kozomo 1-chome, Higashi-ku, Kumamoto-shi, Kumamoto-ken, Japan	32.83685°N; 130.78337°E	Daiqin Li, Bo Wu
*Heptathela kimurai* (Kishida, 1920)	XUX-2013-351	female	Shiroyama Park, Shiroyama-cho, Kagoshima-shi, Kagoshima-Ken, Japan	31.59704°N; 130.55087°E	Daiqin Li, Bo Wu
*Heptathela yanbaruensis* Haupt, 1983	XUX-2014-038A	female	Genka, Nago-shi, Okinawa, Japan	26.62753°N; 128.06044°E	Daiqin Li, Bo Wu
*Liphistius laoticus* Schwendinger, 2013	XUX-2013-267	male	Tad Fane Waterfall, Pakse-Paksong Road, Champasak, Laos	15.18264°N; 106.12713°E	Daiqin Li, Fengxiang Liu, Xin Xu
	XUX-2013-256	female	Tad E-Tu Waterfall, Pakse-Paksong Road, Champasak, Laos	15.19408°N; 106.10161°E	Daiqin Li, Fengxiang Liu, Xin Xu
*Qiongthela baishensis* sp. n.	XUX-2012-087	male	Nangaoling Forest Plantation, Baisha County, Hainan Province, China	19.24934°N; 109.38940°E	Daiqin Li, Fengxiang Liu, Xin Xu
	XUX-2012-089	male	Nangaoling Forest Plantation, Baisha County, Hainan Province, China	19.24934°N; 109.38940°E	Daiqin Li, Fengxiang Liu, Xin Xu
	XUX-2012-092	male	Nangaoling Forest Plantation, Baisha County, Hainan Province, China	19.24940°N; 109.38934°E	Daiqin Li, Fengxiang Liu, Xin Xu
	XUX-2012-086	female	Nangaoling Forest Plantation, Baisha County, Hainan Province, China	19.24934°N; 109.3894°E	Daiqin Li, Fengxiang Liu, Xin Xu
	XUX-2012-085A	female	Nangaoling Forest Plantation, Baisha County, Hainan Province, China	19.24934°N; 109.3894°E	Daiqin Li, Fengxiang Liu, Xin Xu
*Ryuthela nishihirai* (Haupt, 1979)	XUX-2012-302	female	Sheyoshi Park, Shuri, Naha, Okinawa Prefecture, Japan	26.22731°N; 127.71532°E	Hirotsugu Ono, Daiqin Li, Fengxiang Liu, Xin Xu
*Ryuthela ishigakiensis* Haupt, 1983	XUX-2013-228	male	Hirakubo River, Ishigaki island, Okinawa, Japan	24.58864°N; 124.31858°E	Daiqin Li, Bo Wu
*Ryuthela sasakii* Ono, 1997	XUX-2012-364	female	Maja, Nakazato-son, Kumejima Island, Okinawa, Japan	26.35823°N; 126.80168°E	Daiqin Li, Fengxiang Liu, Xin Xu
*Sinothela sinensis* (Bishop & Crosby, 1932), comb. n.	XUX-2012-045	male	Shiqiao Village, Shiqiao Town Yiyuan County, Shandong Province, China	36.15213°N; 118.33400°E	Fengxiang Liu, Zeliang Liu, Xin Xu
	XUX-2012-035	female	Caojia Village, Puji Town, Zhangqiu City, Shandong province, China	36.72776°N; 117.61112°E	Fengxiang Liu, Zeliang Liu, Xin Xu
*Songthela hangzhouensis* (Chen, Zhang & Zhu, 1981), comb. n.	XUX-2013-175	male	Wengjia Village, Mt. Shifeng, Lingyin District, Hangzhou City, Zhejiang Province, China	30.22069°N; 120.11679°E	Daiqin Li, Fengxiang Liu, Zengtao Zhang, Xin Xu
	XUX-2013-170	female	Wengjia Village, Mt. Shifeng, Lingyin District, Hangzhou City, Zhejiang Province, China	30.22074°N; 120.11555°E	Daiqin Li, Fengxiang Liu, Zengtao Zhang, Xin Xu
*Songthela goulouensis* (Yin, 2001), comb. n.	XUX-2011-078*	male	Zizhu Taoist Temple, Hengshan, Hunan Province, China	27.27707°N; 112.70016°E	Fengxiang Liu, Rong Xiao, Xin Xu
	XUX-2011-043	female	Zhonglieci, Hengshan, Henyang, Hunan Province, China	27.27074°N; 112.71507°E	Fengxiang Liu, Rong Xiao, Xin Xu
*Vinathela abca* (Ono, 1999), comb. n.	XUX-2013-048	female	9 KM QL 4D, Coc San, Bat Xat District, Lao Cai Province, Vietnam	22.44414°N; 103.93818°E	Daiqin Li, Fengxiang Liu, Xin Xu
	XUX-2013-049	female	9 KM QL 4D, Coc San, Bat Xat District, Lao Cai Province, Vietnam	22.44414°N; 103.93818°E	Daiqin Li, Fengxiang Liu, Xin Xu
*Vinathela cucphuongensis* (Ono, 1999), comb. n.	XUX-2013-007*	male	Cuc Phuong National Park, Nho Quan, Ninh Binh Province, Vietnam	20.26831°N; 105.69324°E	Daiqin Li, Fengxiang Liu, Xin Xu
	XUX-2013-006	female	Cuc Phuong National Park, Nho Quan, Ninh Binh Province, Vietnam	20.26831°N; 105.69324°E	Daiqin Li, Fengxiang Liu, Xin Xu
